# Exploring the Effects of Land Use Changes on the Landscape Pattern and Soil Erosion of Western Hubei Province from 2000 to 2020

**DOI:** 10.3390/ijerph19031571

**Published:** 2022-01-29

**Authors:** Jiyun Li, Yong Zhou, Qing Li, Siqi Yi, Lina Peng

**Affiliations:** 1 Key Laboratory for Geographical Process Analysis & Simulation of Hubei Province, Central China Normal University, Wuhan 430079, China; realc12@mails.ccnu.edu.cn (J.L.); ennstar@mails.ccnu.edu.cn (Q.L.); siqiyi@mails.ccnu.edu.cn (S.Y.); 2 The College of Urban & Environmental Sciences, Central China Normal University, Wuhan 430079, China; 3 Wuhan Natural Resources and Planning Information Center, Wuhan 430014, China; linapeng@whu.edu.cn

**Keywords:** soil erosion, landscape pattern, land use and land cover change, correlation analysis

## Abstract

Accelerated land use and land cover changes affect regional landscape patterns and change the ecological environment, including soil conservation capabilities. This is not conducive to the sustainable development of human society. In this research, we explored the land use change pattern and landscape change pattern in western Hubei from 2000 to 2020. Using the Chinese soil loss equation and stepwise regression, we measure how landscape patterns affect soil erosion under land use and cover changes in western Hubei Province. The results show that average soil erosion in the mountainous areas of western Hubei tended to increase from 2000 to 2010 and decrease from 2010 to 2020; soil erosion was higher in the western than in the eastern part of the study area. The land in areas with high-intensity and low-intensity soil erosion was mainly waterfront/grassland and cropland/forestland, respectively, and the area of moderate to severe soil erosion was greatest when the slope was 10–20°. When the slope exceeded 20°, the soil erosion area of each grade tended to decrease; thus, 20° is the critical slope for soil erosion in the study area. The landscape pattern in mountainous areas changed dramatically from 2000 to 2020. At the landscape level, landscape fragmentation increased and connectivity decreased, but the area of landscape diversity was stable. Soil erosion in western Hubei was positively correlated with the contiguity index, aggregation index and largest patch index but negatively correlated with the Shannon evenness index. The higher the landscape fragmentation and the greater the accumulation of single land-use types, the more severe the soil erosion is, while the higher the landscape connectivity and the richer the landscape diversity, the less severe the soil erosion is. The results can inform regional landscape management and soil conservation research.

## 1. Introduction

Soil erosion processes have been affected by human activities [1,2]. The increase in soil erosion rates across landscapes can be observed around the world [3,4]. For over a century, the scientific community has been analyzing the soil erosion process [5,6] and dealing with the negative impacts that soil erosion has on the socioenvironment [7,8]. Research on the mechanics of soil erosion has facilitated the study of developing quantitative soil erosion prediction equations based on physical factors such as climate, soil characteristics, vegetation type, and topography [9,10]. Traditional soil erosion prediction methods require large investments in money, time, and field work [11]. The trend of soil erosion prediction methods is to obtain soil erosion prediction results that meet the requirements within a certain accuracy range and with limited data input. With the well-established use of geospatial technologies such as geographic information systems (GIS), spatial interpolation techniques, and the ever-growing range of environmental data, today, research around the world has proposed over 435 distinct soil erosion models, which are playing an increasingly important role in the design and implementation of soil management and conservation strategies [12,13]. Among these models, the universal soil loss equation (USLE) and the revised universal soil loss equation (RUSLE) are the most widely applied models on a global scale [14]. Compared to models based on physical processes (e.g., WEPP and EUROSEM), the USLE has a simpler structure and requires fewer data, so it is widely used for hydroerosion studies at different scales (e.g., intercontinental, national, and state) [14,15]. Therefore, the applications of models belonging to the (R)USLE family are growing [14,16].

In China, the unprecedented urban construction, road and railroad construction and new agricultural and forestry development in the twenty-first century, along with the increase in the intensity of human activities and unreasonable land use, has led to changes in regional topographic conditions, the destruction of vegetation resources and the deterioration of soil characteristics, in turn intensifying soil erosion [3,17,18]. Under this background, the (R)USLE family is also widely used for soil erosion prediction in China to provide scientific references for regional land use management [16]. Considering the unique soil erosion characteristics and long-established erosion control measures of China, Liu Baoyuan established the Chinese soil loss equation (CSLE) based on the revised universal soil loss equation (RUSLE) [19,20]. To gain insight into regional soil erosion characteristics and provide a better reference for regional soil management and land use strategies, scholars applied the CSLE model with geospatial technologies to explore the influence of land use change on soil erosion. Jing Liu and Honghu Liu (2020) calculated the soil erosion modulus of sheet and rill erosion by the CSLE and estimated the amount of gully erosion by gully volume based on a generalized inverted triangular pyramid model multiplied by bulk density [21]. Lin et al. (2020) investigated the spatiotemporal distribution of soil erosion by the CLSE and further quantified the factors influencing soil erosion in the Three Gorges Reservoir Area by GeoDetector [22]. Huang et al. (2020) explored the impact of land use and slope on soil erosion in the Jiuyuangou watershed by using the CSLE [23]. Environmental patterns strongly influence ecological processes [24,25,26]. Anthropogenic activities (e.g., farming, timber harvest) can disrupt the structural integrity of landscapes, which would influence ecological services, including soil conservation, across the landscape [27,28,29,30]. Understanding the influence of landscape pattern change on regional soil erosion could aid in the design of land development plans conducive to soil and water conservation in regional land use layouts [31,32,33]. Although existing researchers have investigated the relationship between land cover change and soil erosion [34,35,36,37], few researchers have explored the relationship between landscape and soil erosion [26,38].

According to the 2019 Hubei Soil and Water Conservation Bulletin, the soil erosion area in Hubei is 32,024.77 km^2^, the soil erosion type is mainly hydraulic erosion, the soil erosion area accounts for 17.22% of the national land area of China, and the erosion intensity is much higher than the allowable intensity [39]. In recent years, with the implementation of the national project of returning farmland to forest and grass (Grain for Green) [40,41] and the soil and water conservation project [42,43], the area of sloping and bare land has been reduced. However, there is still a large amount of soil loss and a large area of loss. Soil erosion leads to a decrease in cropland and thinning of the arable layer, which affects food security [44]. Furthermore, soil erosion causes flash floods and mudslides [45], which affect the economic and social development of mountainous areas [46].

**Figure 1:**
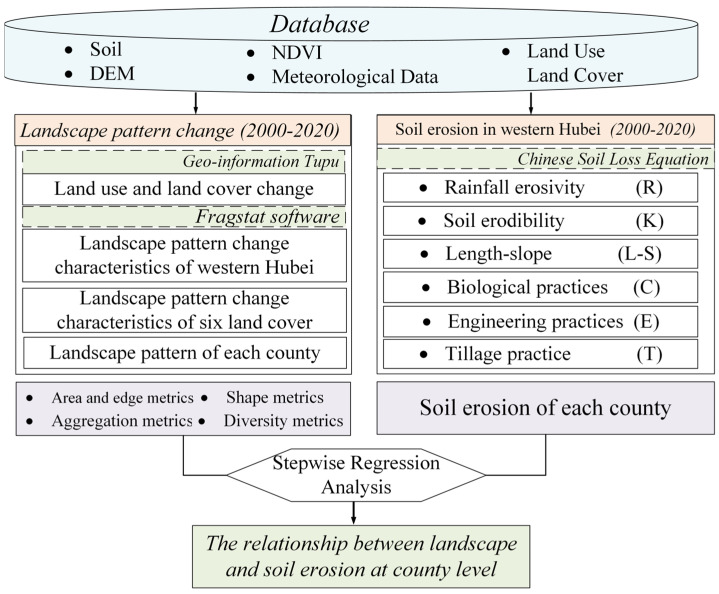
Framework of this study.

Based on the existing research on the improvement of soil erosion prediction models, the soil erosion problems faced in western Hubei Province, and the lack of research on the relationship between regional landscape pattern change and soil erosion in western Hubei. This study attempts to answer the following questions: (1) What are the spatial characteristics of land use change and soil erosion change in western Hubei from 2000–2020? (2) How did the overall landscape of western Hubei change from 2000–2020? How did the landscape patterns of different land covers (cropland, forestland, grassland, water bodies, built-up area, bare) in the study area change? (3) What kinds of landscape pattern characteristics are associated with soil erosion in each county of western Hubei? How relevant is it? To answer question (1), we combined meteorological data, soil data and remote sensing data, using Geo-information Tupu to derive regional land use and land cover change characteristics and using CSLE to calculate regional soil erosion in 2000, 2005, 2010, 2015 and 2020. To answer (2), we used Fragstat 4.2 software to calculate landscape indicators from four perspectives (area and edge, shape, aggregation, diversity characteristics of landscape.). To answer question (3), we combined the results of the soil erosion calculations and the landscape pattern indicator calculations with stepwise regression analysis using SPSS 25 software. This study provides scientific guidance and a decision-making basis for the optimal allocation of land resources and effective control of soil erosion in the mountainous areas of western Hubei Province by understanding the spatial and temporal layout of soil erosion and the relationship between land landscape patterns and soil erosion in each county. The framework of this study is shown in Figure 1.

## 2. Study Area and Data Sources

### 2.1. Study Area

**Figure 2:**
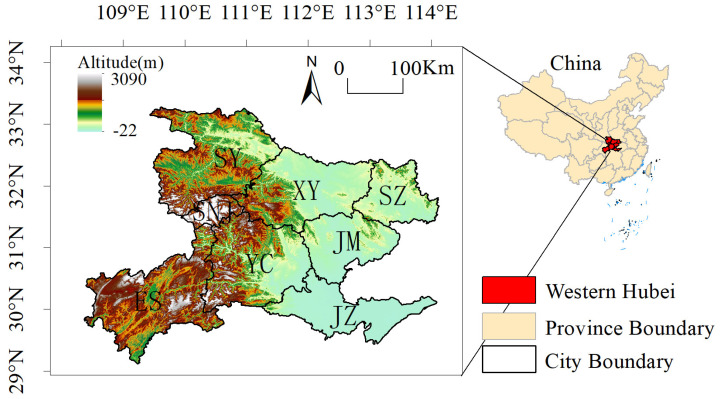
Topographic map of western Hubei Province.

Western Hubei Province is composed of eight cities: Xiangyang (X.Y.), Yichang (Y.C.), Jingzhou (J.Z.), Jingmen (J.M.), Shiyan (S.Y.), Suizhou (S.Z.), Enshi (E.S.), and Shennongjia (S.N.J.) (Figure 2). It includes two provincial subcenter cities, Xiangyang and Yichang, and the only minority autonomous state in Hubei Province, Enshi Tujia and Miao Autonomous Prefecture. The total population and area of the territory account for 50% and 70% of those of Hubei Province, respectively. It is an area rich in ecological and cultural tourism resources in Hubei Province. In the past two decades, urbanization and industrialization have changed regional land cover rapidly. By overlaying China’s land use remote sensing monitoring data in 2000 and 2020, we found that between 2000 and 2020, 26.27% of the cropland in western Hubei Province was converted to forestland, and 5.03% of cropland and 5.06% of the water area were converted to built-up areas, which shows that land use and land cover have dramatically changed, in turn altering the regional ecosystem and ecological services and affecting regional soil conservation.

### 2.2. Data Sources

The data in this research are summarized in Table 1. The land use and land cover (LULC) dataset were derived by the human-computer interactive interpretation method of remotely sensed land cover information to interpret the Landsat TM digital images (Landsat 5 TM (2000, 2005, 2010); Landsat 8 OLI (2015, 2020)) covering China. This dataset includes 6 classes and 25 subclasses of land use. The six classes of land use include cropland, forestland, grassland, water bodies, built-up land, and unused land. The accuracy of the 6 classes of land use was above 94.3% [47,48]. The meteorological dataset includes daily wind direction, daily wind speed, daily precipitation, daily air pressure, and daily temperature [49]. The soil data included the sand, silt, clay, and organic contents of the soil (%) [50]. The annual normalized vegetation index (NDVI) dataset for China was derived from the maximum value of the SPOT/VEGETATION PROBA-V 1 KM PRODUCTS decadal NDVI dataset (http://www.vito-eodata.be (accessed on 10 January 2020)) [48]. ASTER Global Digital Elevation Model (ASTERGDEM) data from the Geospatial Data Cloud (www.giscloud.cn (accessed on 10 January 2020)) [51]. The data were resampled to a resolution of 1 km × 1 km.

## 3. Methods

### 3.1. Geo-Information Tupu

Geo-information Tupu is the visualization of “space and process” research. The map unit codes of the land cover types of the previous two periods are overlayed to record the evolution process of the land use pattern [53].

*C* = 10 × *A* + *B* (1)

where *C* is the map unit grid map that characterizes the evolution characteristics of the land use pattern during the study period. *A* is the map unit grid attribute value of the land use type of the study area in the previous period. *B* is the land use type of the study area in the later period. Based on the remote sensing monitoring data of land cover status, this paper assigns 6 land cover types as 1–6 based on arable land, forestland, grassland, water area, building land and unused land and uses the raster calculator function of ArcGIS 10.7 software to calculate the land use change pattern mapping characteristics of western Hubei Province from 2000–2005–2010–2015–2020.

### 3.2. Chord Diagram Analysis of Land Cover Change

The chord diagram is mainly used to show the relationship between multiple objects [54,55]. The line segment connecting any two points on the circle is called the chord, and the chord (the line between the two points) represents the relationship between the two. The chord diagram can reflect the number of transitions and flow relationships between different territorial spaces in the evolution of territorial space and visualize them. The wider the width of the chord (connecting line), the higher the number of transitions between different territorial spaces. This article uses Power BI software to visualize the transition of different land cover types.

### 3.3. Chinese Soil Loss Equation (CSLE)

We used the CSLE:

(2) A=R⋅K⋅L⋅S⋅B⋅E⋅T

where *A* is the soil loss in t·ha^−1^·yr^−1^. The calculation steps for other factors in this formula are as follows. *R* is the rainfall erosivity in MJ·mm·ha^−1^·yr^−1^. *K* is the soil erodibility in t·h·MJ^−1^·mm^−1^.·*L* and *S* are dimensionless topographic factors of slope length and slope steepness. *B* is the dimensionless vegetation cover factor of biological practices for trees, shrubs, and grasslands. *E* is the dimensionless factor of engineering practices. *T* is the dimensionless factor of tillage practices. Details of the computational steps are given in Li Qing (2021) et al. [56]. The western part of Hubei Province is a typical hydraulic erosion area, and according to the Soil Erosion Classification and Grading Standard (SL190-2007) issued by the Ministry of Water Resources of China, soil erosion is divided into six classes (Table 2).

### 3.4. Landscape Pattern Analysis

We calculated the landscape index to quantify the landscape change pattern from 2000 to 2020 using Fragstat 4.2 software (University of Massachusetts Amherst, Amherst, MA, USA). There are three main types of landscape metrics, including patch-level metrics, class-level metrics, and landscape-level metrics. Patch metrics are defined for individual patches and characterize the spatial character and context of patches. Class metrics are integrated over all the patches of a given type (class). Landscape metrics are integrated over all patch types or classes over the full extent of the data (i.e., the entire landscape) [57]. Our research questions were (1) how the whole landscape in western Hubei changed from 2000 to 2020, (2) how the landscape pattern of each land use and land cover type (cropland, forestland, grassland, water bodies, bare land) changed from 2000 to 2020, and (3) which landscape index had a significant relationship with the soil erosion of each county. Thus, we performed the analysis at the landscape level and class level. The landscape metrics were selected according to the aspect of landscape index measuring and the most commonly used metrics in a previous study [26,58,59] (Table 3). See Appendix A for a brief description of each index (Table A1) [57].

### 3.5. Regression Analysis

Stepwise regression analysis of soil erosion and landscape patterns were conducted by SPSS 25 software to investigate which landscape pattern index influences regional soil erosion. Its essence is to establish the “optimal” multiple linear regression equation. The basic idea of the stepwise regression analysis method is to automatically select the most important variable from available variables to establish a prediction or explanation model for regression analysis [60]. The steps are as follows: add the independent variables to the regression one by one, and check whether the square of the partial regression of the regression equation is significant. After each new independent variable is added, the old independent variables are tested one by one, and the independent variables with insignificant sum of squares of partial regression are eliminated. This continues to be added and removed until neither the new variable is added nor the old variable is deleted.

## 4. Results

### 4.1. Land Cover Change from 2000 to 2020 in Western Hubei Province

**Figure 3:**
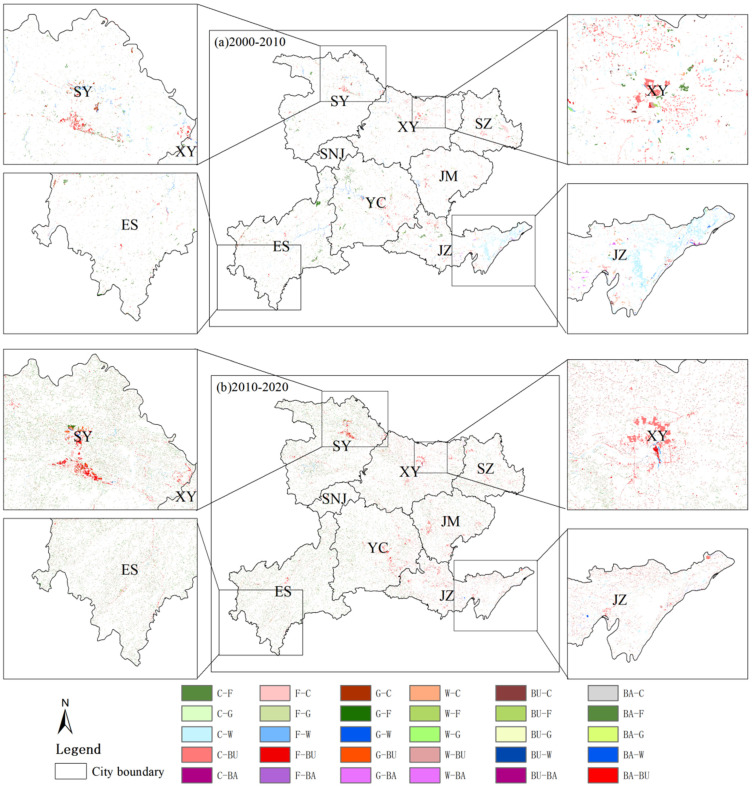
Land cover change in western Hubei Province from (**a**) 2000–2010 and (**b**) 2010–2020 (C—cropland, F—forestland, G—grassland, W—water basin, BU—built-up area, BA—bare; C-F means land cover from cropland to forestland).

**Figure 4:**
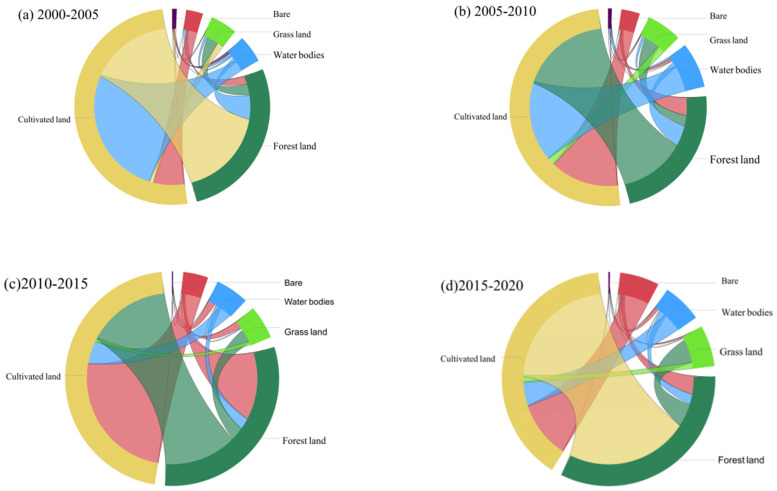
Land cover transition from (**a**) 2000–2005, (**b**) 2010–2010, (**c**) 2010–2015, (**d**) 2015–2020.

From 2000 to 2020, there were 31 types of land use conversion units with temporal heterogeneity (different land use types in different periods) in the study area (Figure 3). The years with the most dramatic change were 2015–2020, with a total area of 9847.15 km^2^. The 31 types of land use conversion units were sorted according to their size, and the sum of the areas of the first 16 types of conversion units accounted for 97.32% of the total conversion area (Figure 4). The most drastically changed land type from 2000 to 2005 was from arable land to water area (C–W in Figure 3), accounting for 29.48% of the total converted area, mainly distributed in the plains of Jingzhou city. The second is from forestland to farmland, which accounts for 20.37% of the total conversion area and is mainly distributed in the sloping farmland around the northwest and southwest forest areas. Then, there is from cropland to built-up area (C–BU in Figure 3), which accounts for 7.03% of the total converted area and is mainly distributed in the urban centers of various cities.

**Figure 5:**
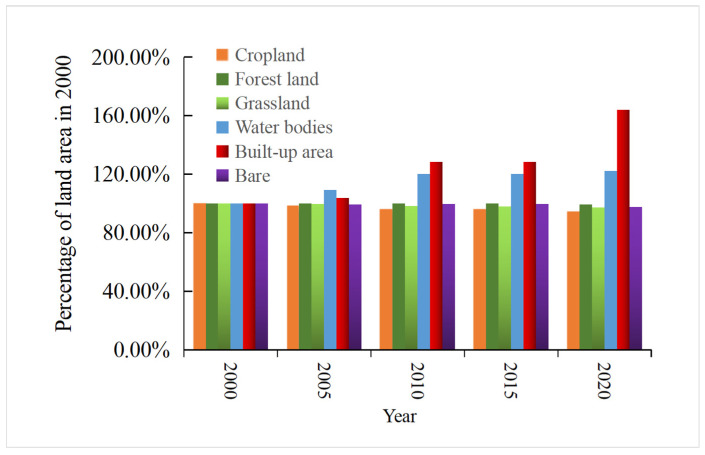
Changes in land cover in western Hubei Province from 2000 to 2020 (Note: The area of each land cover type in 2000 is used as the standard.).

The land use changes from 2000 to 2010 were not as drastic as the land cover changes from 2010 to 2020, but the conversion of cropland to forestland, built-up area, water area, and grassland took place in a large proportion. The 18 types of land use conversion units are sorted by area, and the total area of the first 9 types of conversion accounted for 90.72% of the total conversion area. The most obvious land type conversion is the interchange of cropland and forestland, which accounts for 50.52% of the converted area (Figure 4) and is distributed in the western mountainous area. “Cultivated land to built-up area” (C–BU in Figure 3), which accounts for 11.97% of the total converted area, is mainly distributed in the marginal area of the built-up area where land use changes are relatively drastic. Under the policy of increasing built-up land, the urbanization of western Hubei was fast. The decreased cropland was mainly used for built-up land. Before 2000, driven by the benefits of commercial grains, large areas of ecological land in western Hubei were reclaimed into cropland by local residents [61]. The national government has paid attention to this phenomenon and launched the “Grain for Green” project after 2000. Due to the implementation of the “Grain for Green” Project, in 2020, forestland increased by 6.7% (Figure 5). Among this increased forestland, 79.34% was transformed from cropland. Overall, the land use in western Hubei has undergone major changes from 2000 to 2020, which are mainly concentrated on the mutual conversion between cropland and forestland, grassland, built-up area, and waters. The expansion of built-up land and the “Grain for Green” project in ecologically fragile areas of central China exploited a large amount of cropland [40,62].

### 4.2. Soil Erosion Analysis

#### 4.2.1. Spatial and Temporal Variation in Soil Erosion in Western Hubei Province

**Figure 6:**
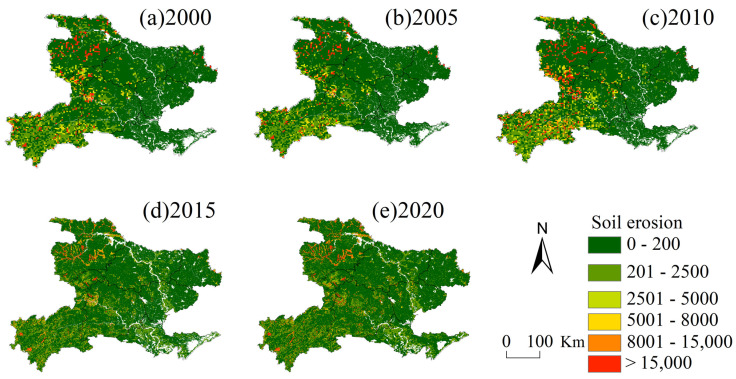
Soil erosion map of western Hubei Province in (**a**) 2000, (**b**) 2005, (**c**) 2010, (**d**) 2015, (**e**) 2020 (t·ha^−1^·yr^−1^).

Based on this classification standard, the soil erosion maps for 2000, 2005, 2010, 2015 and 2020 were created (Figure 6). The average soil erosion in western Hubei Province showed a trend of increasing and then decreasing between 2000 and 2020. Specifically, the soil erosion in western Hubei Province was 3262.52 t ha^−1^ yr^−1^ in 2000, significantly increased in the following 10 years, and gradually reached a peak of 6894.22 t ha^−1^ yr^−1^ in 2010. Thereafter, the soil erosion began to decline, and by 2020, it was only 3140.35 t ha^−1^ yr^−1^. The spatial distribution of soil erosion was consistent from 2000 to 2020, showing an overall pattern of high in the west and low in the east, with the high-value areas mainly located in Shiyan city, the Shenlongjia Forest Area, Yichang city, Enshi Tujia and Miao Autonomous Prefecture and other areas with higher elevations. The soil erosion intensity in western Hubei Province from 2000 to 2020 was mainly slight and mild, but from 2005 to 2010, the areas of intense erosion, very intense erosion and severe erosion were mainly in Shiyan city, Xiangyang city, Jingmen city and other areas with low topography. The Shenlongjia Forest Area and Enshi Tujia and Miao Autonomous Prefecture, with the most significant erosion in the Danjiangkou Reservoir area, showed a decreasing trend after 2010. This was closely related to the construction of the South-to-North Water Diversion Project in China [63]. The Danjiangkou Reservoir of the South-to-North Water Diversion Project was built in 2000, and high-intensity project implementation was not conducive to the regional ecological environment and soil conservation [63]. As a result, erosion expanded in some areas, such as Danjiangkou city. In 2010, when the South-to-North Water Diversion Project was officially completed, China took a series of measures to restore the ecological environment in areas along the route. In 2013, the Hubei Provincial Party Committee proposed actively promoting the Yangtze River Ecological and Economic Belt and the Han River Ecological and Economic Belt; the ecological environment in the western part of Hubei Province improved, and soil erosion was subsequently reduced [64].

**Figure 7:**
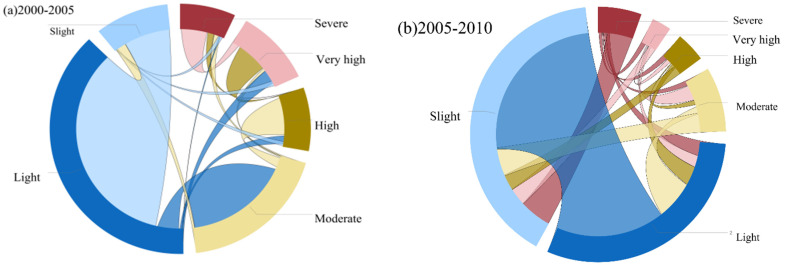

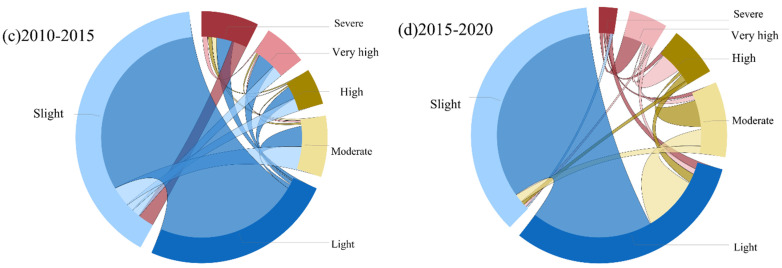
Transition of land at different soil erosion level from (**a**) 2000–2005, (**b**) 2010–2010, (**c**) 2010–2015, (**d**) 2015–2020.

Between 2000 and 2020, the percentage of land with a light grade becoming a slight erosion grade was the highest in the area of land with a change in soil erosion grade, 37.55% for the years 2000–2005, 18.75% for the years 2005–2010, 35.11% for 2010–2015, and 36.73% for 2015–2020. This is followed by a larger area of land with light grade becoming slight erosion grade with 7.9%, 18.75%, 25.23%, and 20.93%, respectively. The increase in soil erosion areas between 2005 and 2010 is reflected in the increase in land change from slight to severe, accounting for 5.51% of the total. Between 2015 and 2020, soil erosion increased, as evidenced by more land eroding from moderate to high levels of soil erosion, accounting for 4.77% of all (Figure 7).

#### 4.2.2. Land Cover and Soil Erosion

**Figure 8:**
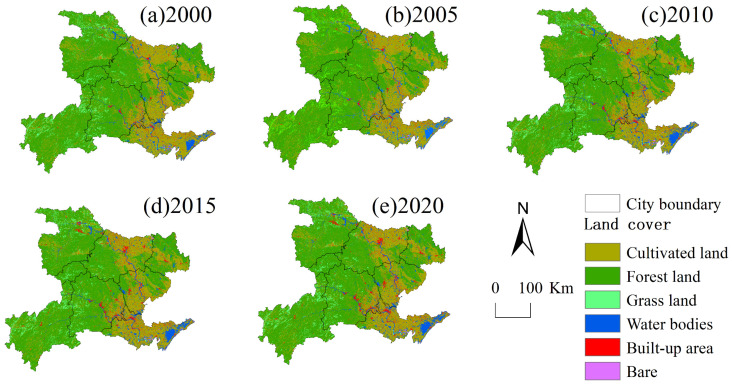
Land use map of western Hubei Province in (**a**) 2000, (**b**) 2005, (**c**) 2010, (**d**) 2015, (**e**) 2020.

The land cover type in the high-intensity soil erosion area is mainly waterfront and grassland, while that in the low-intensity soil erosion area is mainly cropland and forestland. Grassland areas are mainly grazing areas with poor vegetation cover, weak vegetation interception and infiltration ability, serious soil erosion and high soil erosion intensity. Waterfront areas are affected by river erosion and soil erosion, coupled with poor vegetation growth environments along rivers, poor soil and water conservation abilities and high soil erosion. The soil erosion intensity is lower because forestland is densely vegetated and has high coverage and good soil and water conservation abilities, while cropland has been impacted by soil and water protection projects and is generally located in areas with low terrain, resulting in less rainwater scouring [65] (Figure 8).

**Figure 9:**
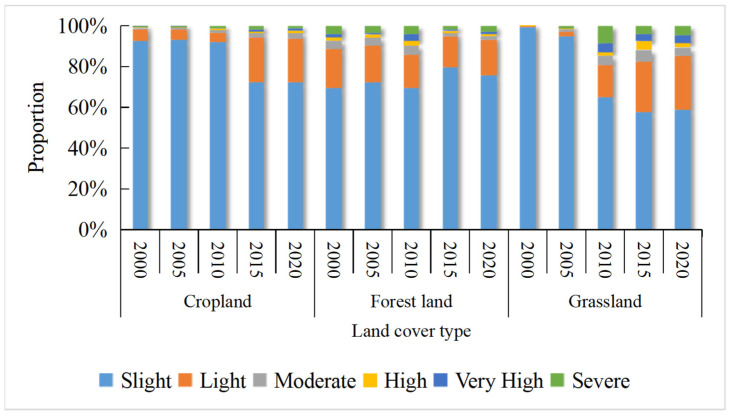
Proportion of area of different soil erosion classes in different land types (cropland, forestland, grassland).

Since the built-up area and water area have soil erosion values of 0 and are considered slightly eroded, cropland, forestland and grassland are selected as the objects to analyze the relationship between soil erosion and land use (Figure 9). From 2000–2020, the soil of each land use type in western Hubei Province mainly experienced light erosion, and the area of light erosion accounted for more than 55% of all of the land area. In cropland, the area of very strong erosion was smallest, while that of slight erosion was largest and showed a significant decrease from 92.4% in 2000 to 72.22% in 2020. The areas of light, moderate, strong, very strong and severe erosion showed an overall increase, with the area of light erosion expanding the most: a total increase of 15.9% from 2000 to 2020.

The areas of soil erosion at different levels showed fluctuating decreases during 2000–2020, while the area of strong erosion showed a trend of first increasing and then decreasing. The areas of strong, intensive, and severe erosion all reached their peaks in 2010, with proportions of 2.40%, 3.02% and 4.29%, respectively, indicating that the soil erosion of forestland improved. Grassland covers a smaller area than cropland and forestland and is dominated by slight and mild erosion, and the area of slight erosion decreased by as much as 40.67%, while the areas of other erosion types showed an increasing trend, with mild erosion showing the fastest growth rate of 95.56%. In 2010, the proportions of very strong erosion and severe erosion in grassland were relatively large, reaching a peak in 20 years, at 4.52% and 8.47%, respectively.

#### 4.2.3. Soil Erosion Analysis at Different Slope Levels

**Figure 10:**
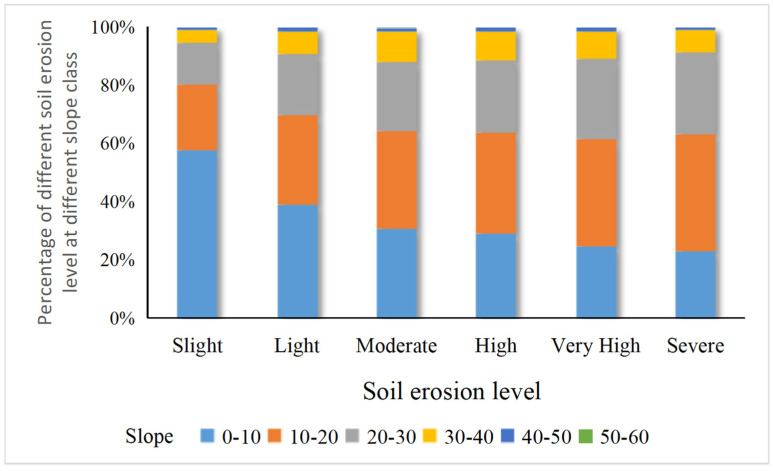
Percentage of soil erosion area in different slope classes in western Hubei Province from 2000–2020.

Topographic factors are among the important factors affecting the intensity of soil erosion, and slope, as the main topographic factor, also has an important influence on soil erosion intensity. In this research, the slope map of western Hubei Province was extracted from the DEM, the slope range was divided into 10° intervals, and the area and proportion of each soil erosion class in different slope ranges were calculated (Figure 10).

The effect of slope differs among soil erosion levels; slight and light soil erosion are the main soil erosion classes at each slope level, and the area of land with slight and light soil erosion shows a decreasing trend with an increase in slope. The area of the other soil erosion levels tends to increase and then decrease with increasing slope, and all of them reach a peak at 10–20°. In the range of 0–10°, soil erosion is mainly slight erosion with an area proportion of 44.90%, followed by light erosion (5.77%). The combined representation of the other soil erosion intensity levels is less than 1%, while the area of moderate to severe soil erosion reaches the maximum when the slope is 10–20°. The area of severe soil erosion is the largest, with a proportion of 0.94%. However, light and slight erosion still dominate in the slope range of 10–20°. When the slope is greater than 20°, the soil erosion area of all grades shows a decreasing trend, which indicates that 20° is the critical slope value for soil erosion in western Hubei Province. When the slope is greater than 50°, high-level soil erosion disappears, and the representation of a low-intensity soil erosion area is less than 0.1%.

### 4.3. Analysis of Landscape Patterns in Western Hubei Province

#### 4.3.1. Landscape Pattern Index Analysis

**Figure 11:**
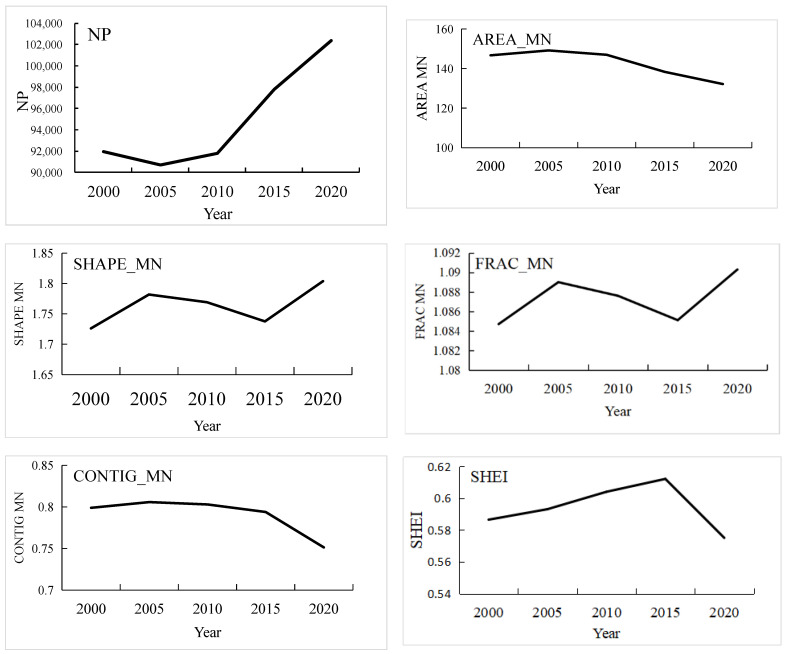
Landscape Pattern Index for Western Hubei Province in 2000, 2005, 2010, 2015, 2020. (CONTIG—Contiguity Index; FRAC—Fractal Dimension Index; NP—Number of Patches; SHEI—Shannon’s Evenness Index).

NP and AREA_MN characterize the degree of regional fragmentation; the larger the NP value or the smaller the AREA_MN value is, the greater the regional landscape fragmentation. The NP value of western Hubei Province significantly increased from 91,977 in 2000 to 102,373 in 2020, and the AREA_MN value showed an overall decrease of 9.88%, indicating that the landscape fragmentation of western Hubei Province has increased. SHAPE_MN and FRAC_MN indicate the shape of the patches, and these two values showed a slight increase in western Hubei Province from 2000 to 2020, indicating that the overall change in the shape of landscape patches in western Hubei Province was small. The FRAC_MN value is greater than 1, which means that the regional landscape is influenced more by humans, but the influence shows a decreasing trend. CONTIG_MN shows a decreasing trend, and the degree of connectivity between regional landscape patches decreases, which indicates that the landscape in western Hubei Province was more dispersed and patchily distributed during 2000–2020 (Figure 11).

The SHEI is used to measure the heterogeneity of the landscape in the region, with the magnitude of the SHEI value indicating the uniformity of landscape types in the region. The SHEI value fluctuated during 2000–2020, showing an increase from 2000 to 2015 and a decrease thereafter, but the changes in the value did not exceed 0.004. Moreover, the SHEI values were all greater than 0.57, indicating that in western Hubei Province, the overall distribution of landscape patches was relatively uniform, and landscape diversity tended to be stable, but there were still some changes in different periods.

#### 4.3.2. Landscape Pattern Index Analysis for Each Land Use Cover Type

**Figure 12:**
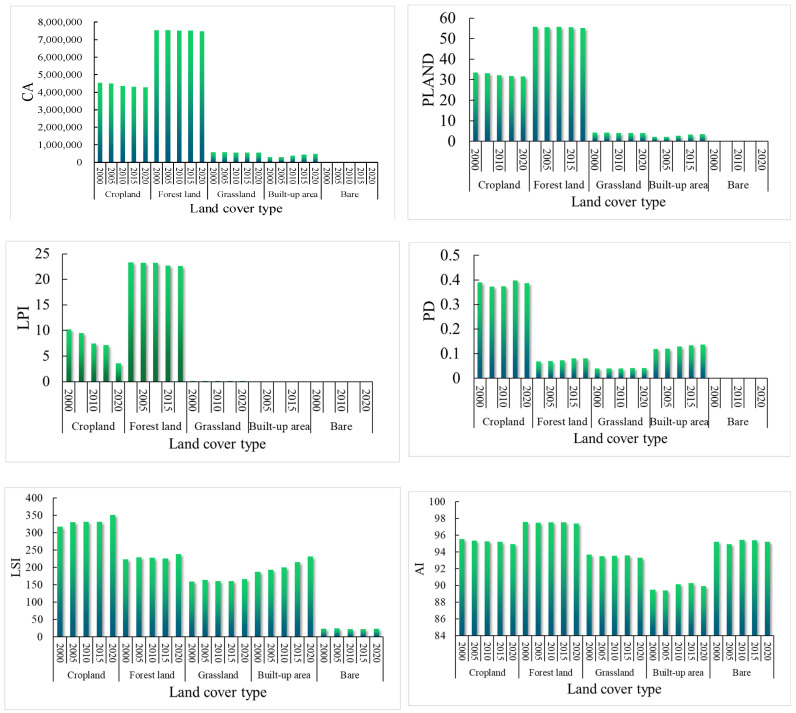
Landscape pattern index analysis for cropland, forestland, grassland, water bodies, bare land (CA—Class area; PLAND—Percent of landscape; LPI—Largest patch index; PD—Patch density; ED—Edge density; LSI—Landscape shape index; AI—Agglomeration index).

Figure 12 shows the landscape pattern indices at the class level in western Hubei Province, and the trends of different indices vary among years and land types. In terms of class area (CA), the areas of cropland, forestland, grassland, and unused land decreased, with that of forestland decreasing the most, while only the area of built-up land showed an increasing trend, with an increase rate of 64.07%. The percentage of landscape (PLAND) and largest patch index (LPI) are consistent with the temporal change trend of CA for different land use types. The PLAND and LPI values of cropland and forestland are the largest, while those of unused land and built-up area are relatively low, which indicates that cropland and forestland are the dominant land types in the region and that the dominance of unused land and built-up area is lower (Figure 12). The detailed value of Landscape pattern index analysis for each land cover type is in Appendix A, Table A2.

PD refers to the number of patches per unit area, and its magnitude characterizes the fragmentation of the landscape in the region. The PD value of cropland is higher, approximately 0.38, than that of other land types due to the influence of human production and living behavior and rapid urbanization, and the fragmentation degree is higher. In addition, the changes in the PD values of each land use type from 2000 to 2020 were small, indicating that the landscape patches in the region were relatively stable. Among all land use types, except for unused land, the edge density (ED) and landscape shape index (LSI) showed fluctuating increases, and the LSI value was higher, indicating an increase in landscape edge heterogeneity in the region. The patches of all land types except unused land show a complex shape with high boundary fragmentation, but the LSI of the built-up area increases significantly, while the LSI values of other land types show small temporal changes. The aggregation index (AI) reflects the degree of aggregation of the landscape patches in a region. The AI values of each land use type have small changes and high values, indicating that the landscape distribution areas in the region are stable and have a high degree of aggregation (Figure 12).

The CONTIG_MN, LPI and SHEI passed the significance test at *p* < 0.1, and the AI and PD passed the significance test at *p* < 0.05 (Table 4). Soil erosion in western Hubei Province shows a positive correlation with CONTIG_MN, AI, and LPI. When the average connectivity of the landscape in the region increases, the similarity between patches is stronger, the landscape diversity decreases, and the ability to maintain regional soil and water is low. Similarly, the increases in the aggregation index and the maximum patch index indicate that the dominance and aggregation of patches increase. This leads to an increase in the proportion of a single landscape in the region; a single landscape has a weaker ability to maintain soil, and soil erosion is therefore high. In addition, soil erosion in western Hubei Province showed a negative correlation with the SHEI, which is an important index for comparing changes in diversity across landscapes or in the same landscape over time. When the SHEI value is high, the higher the landscape heterogeneity is, the stronger the soil and water conservation abilities, and the higher the ability to effectively prevent the intensification of soil erosion. Conversely, the landscape dominance is relatively high, the landscape abundance is reduced, the soil conservation ability is weaker, and the soil erosion is high.

## 5. Discussion

### 5.1. Possible Reasons for Soil Erosion Changes in Western Hubei

**Figure 13:**
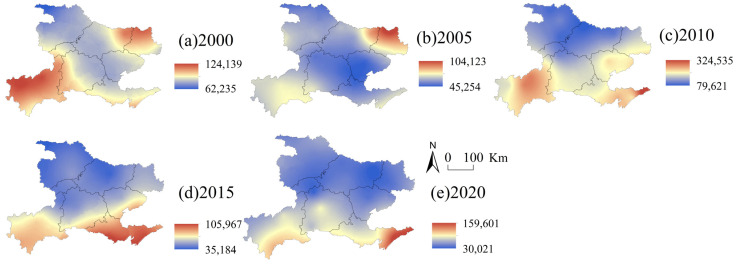
Rainfall erosivity map for western Hubei Province in (**a**) 2000, (**b**) 2005, (**c**) 2010, (**d**) 2015, (**e**) 2020.

The western region of Hubei Province is at the confluence of Daba Mountain and Wushan Mountain, and soil erosion has become one of the main ecological problems restricting the development of local cities. This is consistent with the conclusion of scholars who have investigated soil erosion in Hubei Province [66]. Rainfall is one of the main reasons for the fluctuation in soil erosion in western Hubei. Due to the natural geography of western Hubei Province, soil erosion in the region is mainly hydraulic and gravity erosion, which has been explained in Wang et al.’s (2016) research [67]. In this study, we used the formula of daily rainfall erosion force to analyze the regional rainfall erosion force in western Hubei Province (Figure 13). The lowest rainfall erosion force in 2020 was approximately 1 time lower than that in 2000, indicating that the soil loss caused by precipitation erosion in some areas has improved. In terms of spatial distribution, during 2000–2005, the high values of rainfall erosion force were mainly distributed in the northeastern and southwestern parts of western Hubei Province, mainly in Suizhou city and Enshi Tujia and Miao Autonomous Prefecture, and the low values were mainly distributed in the central part of western Hubei Province, while from 2010, the high values of rainfall erosion force in western Hubei Province gradually shifted to the south, especially in the Danjiangkou Reservoir area, where the rainfall was significantly reduced. In conclusion, the spatial and temporal variation in the rainfall erosion force in western Hubei Province is generally consistent with the variation in the soil erosion rate, which indicates that rainfall is one of the main factors causing soil loss in western Hubei Province.

Land cover is also one of the main factors affecting the amount of soil erosion. As the source of material transport related to human activities and the natural environment, the erosion of soil is influenced not only by natural factors but also by human activities. The area of cropland, grassland, forestland, and unused land in western Hubei Province decreased from 2000 to 2020 (Figure 3 and Figure 4), while the area of watershed and built-up area showed different degrees of increase. Moreover, the rate of change in land use types was relatively large from 2005 to 2010 and from 2015 to 2020 (Figure 13), which was related to the completion of the South-to-North Water Diversion Project, the Hanjiang Ecological and Economic Belt and the Yangtze Ecological and Economic Belt. Importantly, this was related to the completion of the South-to-North Water Diversion Project [63], the construction of the Han River Ecological and Economic Zone and the Yangtze River Ecological and Economic Zone [68], as well as the rapid development of the Yijing-Jing’en City Cluster and the Xiangshi-Shuishen City Cluster [69]. This finding is consistent with the research of Liu et al. (2014) and Ning et al. (2018) [47,61]. The lost cropland was mainly transformed into forestland and built-up areas, while forestland was mainly transformed into cropland and grassland (Figure 3 and Figure 4). The transfer-in transfer-out rate of cropland was high, and its ED and LSI were the highest among all categories with high landscape fragmentation. However, the cropland area plays a protective role against soil erosion through human implementation of various soil and water conservation measures, and the amount of soil erosion is relatively small, while the CA, LPI and PLAND are all at high levels, with good vegetation cover, high landscape integrity and less soil erosion (Figure 11). This finding is similar to the finding of Mirghaed et al. (2018) [58]. In Mirghaed et al.’s (2018) study, cropland with high values of LPI and PLAND had low soil erosion.

In addition, changes in national policies are among the important reasons for the spatial and temporal changes in soil erosion, especially during the construction period of the South-to-North Water Diversion Project. When ecological damage was severe, vegetation cover was reduced, soil and water conservation capacities decreased, and soil erosion increased dramatically [63]. With the completion of the project and the restoration of ecological functions, the ecological environment improved, and the soil erosion problem decreased, which is consistent with the findings of this paper for western Hubei Province [65]. Soil erosion peaked in approximately 2010. In 2018, the National Development and Reform Commission of the People’s Republic of China released the Development Plan for the Han River Ecological and Economic Belt [64]. The ecological environment in western Hubei Province has improved, the stability of the ecosystem has been enhanced, and soil erosion has been reduced. As Hancock et al. (2019) mentioned, if grass cover is reduced under trees, it will leave soil exposed and increase erosion risk. In addition, Ruiz-Colmenero et al. (2013) found that planting vegetative cover crops between rows of vines in sloping vineyards can reduce losses from erosion and improve the infiltration of water [70]. It is necessary to adopt reasonable grazing methods in grasslands to prevent soil erosion caused by overgrazing [71,72] and take measures to increase vegetation cover and strengthen soil and water conservation projects in western Hubei Province.

### 5.2. Comparison with Existing Research

**Figure 14:**
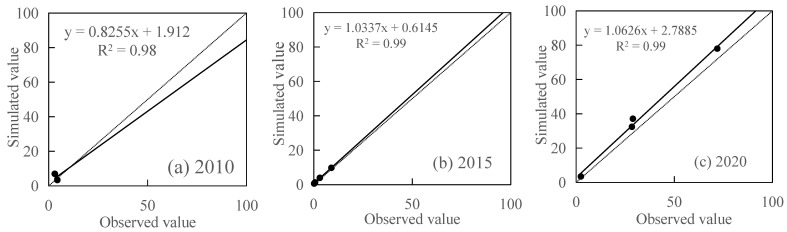
Correlation analysis between simulated value (10^5^ t) and observed value (10^5^ t) in (**a**) 2010, (**b**) 2015 and (**c**) 2020.

Due to the lack of field erosion data in the region, the soil erosion results estimated by the CSLE model in this study were analyzed using the observation data of river sand transport monitoring stations at the outlet of each watershed of typical rivers (3 river sand transport monitoring stations in 2010 and 2015, 4 river sand transport monitoring stations in 2020) from the Hubei Soil and Water Conservation Bulletin for correlation analysis. The results showed that the overall correlation coefficient was 0.98, 0.99, 0.99 in 2010, 2015 and 2020 (Figure 14), respectively, indicating that the soil erosion estimation results for these 3 years were satisfactory.

This study found that the land use types in the high-intensity soil erosion area are mainly waterfront and grassland, which is consistent with the findings of Wang et al. (2016) [67] and Xu et al. (2017) [73]. However, this finding contrasts with Ouyang et al.’s (2010) finding [26]. In Ouyang et al.’s (2010) study, the erosion intensity of grassland was generally lower than that of cropland. Studies have shown that when grassland is degraded and desertified, its soil erosion rate will be higher than that of crops. The reasons for the different study conclusions may be the different study areas, study years, and soil erosion models. The study area is in the Longliu watershed, which is located at the conjunction of the Qinghai-Tibet and Loess Plateau. The soil physicochemical properties in the Loess Plateau region are different from those in the soil of mountainous regions in the middle reaches of the Yangtze River, and the precipitation characteristics are different. Ouyang et al.’s (2010) study focused on 1977 to 2006. This research was conducted from 2000 to 2020, and the national development policies in those years were different, so the land use activities were different. This study used the CSLE model, while the study of Ouyang et al. used the SWAT model, employing different model parameters and calculation methods to calculate regional soil erosion. In addition, the findings of this study show that the soil erosion intensity in Hubei from 2000 to 2020 was dominated by slight light (Figure 4), which is consistent with Wang et al.’s (2016) [67] research on soil erosion in China.

The results of this study show that the soil erosion intensity in the mountainous areas of western Hubei is positively correlated with CONTIG_MN, AI, and LPI and negatively correlated with SHEI; that is, the fragmentation, diversity, and aggregation of the landscape are closely related to regional soil erosion. These findings are consistent with Ouyang et al.’s (2010) findings [26]. We also compared our research findings with Mirghaed et al.’s (2018) research [58]. Mirghaed et al. (2018) explored the relationship between soil erosion and landscape metrics of different land use types in the Gorgan Watershed in northern Iran. In the results of their study, the trends of the NP and LSI metrics for all land uses can affect the sediment retention and sediment yield. However, in our study, no such correlation was found. Due to different evaluation units, research scales, land cover, and regional natural conditions, there are differences in the correlation between landscape patterns and soil erosion. Our study only took counties as the evaluation unit, calculated the land landscape pattern index and soil erosion amount of each county, and explored the correlation between these two factors. Mirghaed et al. (2018) focused on the relationship of the landscape pattern of each land cover with soil erosion. Since there are still few studies on the correlation between landscape patterns and soil erosion, especially in central China, there are few research results we could compare.

### 5.3. Strengths and Limitations

This study used meteorological station data, soil data and remote sensing data with high data credibility and fills a knowledge gap in the study of the relationship between landscape patterns and soil erosion in western Hubei Province by using the generally accepted method [15,60,74]. In addition, we explored the influence of changing landscape patterns on soil erosion in the western region of Hubei Province from the perspective of the joint influence of natural and human social systems, which is scientific and can be replicated in other regions for comparison.

However, there are still some limitations. First, although landscape factors and external influences such as rainfall, slope, vegetation cover and land use type are considered, the heterogeneity of each factor is not taken into account in the evaluation [15]. In addition, the correlation between landscape patterns and soil erosion at different scales was not examined; for example, the relationship between landscape patterns and soil erosion at the municipal or village level was not investigated. The soil erosion process is a multiscale nonlinear and complex process [75]. In the future, researchers could focus on not only improving evaluation accuracy by modifying the evaluation method of landscape indices and by enhancing the resolution of data but also exploring the scale effects of landscapes on soil erosion. The correlation between landscape patterns and soil erosion at multiple scales can be analyzed in subsequent studies to provide a scientific basis for administrative land use management at different levels.

## 6. Conclusions

In this research, we found that (1) the landscape pattern in mountainous areas dramatically changed from 2000 to 2020. The years with the most dramatic change were 2015–2020, with a total area of 9847.15 km^2^. The most obvious land type conversion is the interchange in cropland and forestland, which accounts for 50.52% of the converted area. (2) The average soil erosion in western Hubei Province showed a trend of increasing from 2000 to 2010, with a peak value of 6894.22 t·ha^−1^·yr^−1^ in 2010, and then decreasing from 2010–2020, with the lowest soil erosion value, 3140.35 t·ha^−1^·yr^−1^, in 2020. Soil erosion was higher in the western than in the eastern part of the study area. The land in areas with high-intensity and low-intensity soil erosion was mainly waterfront/grassland and cropland/forestland, respectively, and the area of moderate to severe soil erosion was greatest when the slope was 10–20°. 20° is the critical slope for soil erosion in the study area because when the slope exceeded 20°, the soil erosion area of each grade tended to decrease. (3) At the landscape level, landscape fragmentation increased and connectivity decreased, but the area of landscape diversity was stable. Soil erosion in western Hubei was positively correlated with the contiguity index, aggregation index and largest patch index but negatively correlated with the Shannon evenness index. The higher the landscape fragmentation and the greater the accumulation of single land-use types, the more severe the soil erosion is, while the higher the landscape connectivity and the richer the landscape diversity, the less severe the soil erosion is. We suggest that future land management in each county of western Hubei should place greater emphasis on landscape connectivity and landscape diversity to reduce land fragmentation and thus soil erosion.

## Figures and Tables

**Table 1:** Data description.

Data Name	Data Source	Time	Units/Resolution
Depth to bedrock map of China	Scientific data [52]	2018	100 m × 100 m
Soil data	Harmonized World Soil Database (HWSD) [50]	2012	1000 m × 1000 m
Land-use/land cover data	Resource and Environment Science and Data Center [48]	2000; 2005; 2010; 2015; 2020	30 m × 30 m
Normalized difference vegetation index			1000 m × 1000 m
Meteorological data	Meteorological Data Center of China Meteorological Administration [49]	2000–2020	Daily
Digital elevation model	Geospatial Data Cloud [51]	2008	30 m × 30 m

**Table 2:** The standard for soil erosion level.

Soil Erosion Level	Slight	Light	Moderate	High	Very High	Severe
Soil erosion rate (t·ha^−1^·yr^−1^)	<200	200–2500	2500–5000	5000–8000	8000–15,000	>15,000

**Table 3:** Landscape index selected at class-level analysis, landscape-level analysis, and regression analysis.

	Landscape Metrics	Landscape-Level Metrics	Class-Level Metrics	Regression Analyze
The Aspect of Landscape Pattern Measured				
Area and edge		AREA_MN	CA; PLAND; ED; LPI	LPI
Shape		SHAPE_MN; CONTIG_MN; FRAC_MN		FRAC_MN; CONTIG_MN
Aggregation		NP	AI; PD	AI; PD; CONTAG; LSI; NP
Diversity		SHEI		SHDI; SHEI

Note: AI—Aggregation Index; CA—Class Area; CONTIG—Contiguity Index; CONTAG—Contagion; ED—Edge Density; FRAC—Fractal Dimension Index; LPI—Largest Patch Index; LSI—Landscape Shape Index; NP—Number of Patches; PLAND—Percentage of Landscape; PD—Patch Density; SHDI—Shannon’s Diversity Index; SHEI—Shannon’s Evenness Index.

**Table 4:** Regression model parameters of the landscape pattern index and soil erosion.

Landscape Pattern Index	Standard Coefficient	Significant Coefficient
CONTIG_MN	0.325	0.071
AI	1.021	0.001
LPI	0.245	0.072
SHEI	−0.411	0.092

Note: AI—Aggregation Index; CONTIG_MN – Mean value of Contiguity Index; LPI—Largest Patch Index; SHEI—Shannon’s Evenness Index.

## Data Availability

Not applicable.

## Appendix A

**Table A1:** Brief description of landscape index in this research (refer Fragstat 4.2 help document [57]).

Metrics Groups	Index	Formula		Range	Comments
Area and edge metrics	Class Area (CA)	$\mathrm{CA}=\sum_{n}^{j=1}a_{\mathrm{ij}}\left(\frac{1}{10,000}\right)$	a_ij_ = area (m^2^) of patch ij.	CA > 0, without limit. CA approaches 0 as the patch type becomes increasing rare in the landscape. CA = TA when the entire landscape consists of a single patch type; that is, when the entire image is comprised of a single patch.	Class area is a measure of landscape composition; specifically, how much of the landscape is comprised of a particular patch type.
	Percentage of Landscape (PLAND)	$\mathrm{PLAND}=P_{i}=\frac{\sum_{n}^{j=1}a_{\mathrm{ij}}}{A}(100)$	P_i_ = proportion of the landscape occupied by patch type (class) i. a_ij_ = area (m^2^) of patch ij. A = total landscape area (m^2^).	0 < PLAND < 100 PLAND approaches 0 when the corresponding patch type (class) becomes increasingly rare in the landscape. PLAND = 100 when the entire landscape consists of a single patch type; that is, when the entire image is comprised of a single patch.	Percentage of landscape quantifies the proportional abundance of each patch type in the landscape.
	Edge Density (ED)	$\mathrm{ED}=\frac{\sum_{m}^{k=1}e_{\mathrm{ik}}}{A}(10,000)$	e_ik_ = total length (m) of edge in landscape involving patch type (class) i; includes landscape boundary and background segments involving patch type i. A = total landscape area (m^2^).	ED ≥ 0, without limit. ED = 0 when there is no class edge in the landscape; that is, when the entire landscape and landscape border, if present, consists of the corresponding patch type and the user specifies that none of the landscape boundary and background edge be treated as edge.	Edge density is a measure of edge length of a particular patch type. ED perform better than Total Edge metric.
	Largest Patch Index (LPI)	$\mathrm{LPI}=\frac{\overset{j=1}{\underset{n}{\max}}\left(a_{\mathrm{ij}}\right)}{A}(100)$	a_ij_ = area (m^2^) of patch ij. A = total landscape area (m^2^).	0 < LPI < 100 LPI approaches 0 when the largest patch of the corresponding patch type is increasingly small. LPI = 100 when the largest patch comprises 100% of the landscape.	Largest patch index at the class level quantifies the percentage of total landscape area comprised by the largest patch.
	AREA	$\mathrm{AREA}=a_{\mathrm{ij}}\left(\frac{1}{10,000}\right)$	a_ij_ = area (m^2^) of patch ij.	AREA ≥ 0	Metrics based on the mean patch characteristic, such as Mean patch size (AREA_MN) or Mean patch shape index (SHAPE_MN), provide a measure of central tendency in the corresponding patch characteristic across the entire landscape.
Shape metrics	SHAPE	$\mathrm{SHAPE}=\frac{0.25p_{\mathrm{ij}}}{\sqrt{a_{\mathrm{ij}}}}$	p_ij_ = perimeter (m) of patch ij. a_ij_ = area (m^2^) of patch ij.	SHAPE ≥ 1, without limit. SHAPE = 1 when the patch is square and increases without limit as patch shape becomes more irregular. SHAPE measures the complexity of patch shape compared to a standard shape (square) of the same size.	
	Contiguity Index (CONTIG)	$\;\mathrm{CONTIG}=\frac{\left[\frac{\sum_{r=1}^{z}c_{\mathrm{ijr}}}{a_{\mathrm{ij}}}\right]-1}{v-1}$	C_ijr_ = contiguity value for pixel r in patch ij. v = sum of the values in a 3-by-3 cell template (13 in this case). a_ij_ = area of patch ij in terms of number of cells.	0 < CONTIG < 1	Contiguity index assesses the spatial connectedness, or contiguity, of cells within a grid-cell patch to provide an index on patch boundary configuration and thus patch shape.
	Fractal Dimension Index (FRAC)	$\mathrm{FRAC}=\frac{2\ln\left(0.25p_{\mathrm{ij}}\right)}{\ln a_{\mathrm{ij}}}$	p_ij_ = perimeter (m) of patch ij. a_ij_ = area (m) of patch ij.	1 < FRAC < 2 FRAC approaches 1 for shapes with very simple perimeters such as squares, and approaches 2 for shapes with highly convoluted, plane-filling perimeters.	Fractal dimension index is appealing because it reflects shape complexity across a range of spatial scales (patch sizes).
Aggregation metrics	Aggregation Index (AI)	$\mathrm{AI}=\left[\frac{g_{\mathrm{ii}}}{\max\to g_{\mathrm{ii}}}\right](100)$	g_ii_ = number of like adjacencies (joins) between pixels of patch type (class) i based on the singlecount method. max-g_ii_ = maximum number of like adjacencies (joins) between pixels of patch type (class) i based on the single-count method.	0 < AI < 100 Given any P_i_, AI equals 0 when the focal patch type is maximally disaggregated; AI increases as the focal patch type is increasingly aggregated and equals 100 when the patch type is maximally aggregated into a single, compact patch.	
	Landscape Shape Index (LSI)	$\mathrm{LSI}=\frac{0.25\sum_{m}^{k=1}e_{\mathrm{ik}}^{*}}{\sqrt{A}}$	$e_{\mathrm{ik}}^{*}$ = total length (m) of edge in landscape between patch types(classes) i and k; includes the entire landscape boundary and some or all background edge segments involving class i. A = total landscape area (m).	LSI > 1, without limit. LSI = 1 when the landscape consists of a single square patch of the corresponding type; LSI increases without limit as landscape shape becomes more irregular.	The Landscape shape index (LSI) index measures the perimeter-to area ratio for the landscape as a whole. The greater the value of LSI, the more dispersed are the patch types.
	Contagion (CONTAG)	$\;\mathrm{CONTAG}=\left[1+\frac{\left[\sum_{m}^{i=1}\sum_{m}^{k=1}\left[P_{i}^{}\frac{g_{\mathrm{ik}}}{\sum_{m}^{k=1}g_{\mathrm{ik}}}\right]\cdot \left[\ln\left(P_{i}^{\circ }\frac{g_{\mathrm{ik}}}{\sum_{m}^{k=1}g_{\mathrm{ik}}}\right]\right]\right.}{2\ln(m)}\right](100)$		P_i_ = proportion of the landscape occupied by patch type (class) i. g_ik_ = number of adjacencies (joins) between pixels of patch types (classes) i and k based on the double-count method. m = number of patch types (classes) present in the landscape, including the landscape border if present.	CONTAG approaches 0 when the patch types are maximally disaggregated and interspersed. CONTAG = 100 when all patch types are maximally aggregated.
	Number of Patches (NP)	$\mathrm{NP}=n_{i}$	N = total number of patches in the landscape.	NP > 1, without limit. NP = 1 when the landscape contains only 1 patch.	Number of patches often has limited interpretive value by itself because it conveys no information about area, distribution, or density of patches.
	Patch Density (PD)	$\mathrm{PD}=\frac{n_{i}}{A}(10,000)(100)$	N = total number of patches in the landscape. A = total landscape area (m^2^).	PD > 0, constrained by cell size.	Patch density has the same basic utility as number of patches as an index, except that it expresses number of patches on a per unit area basis that facilitates comparisons among landscapes of varying size.
Diversity metrics	Shannon’s Diversity Index (SHDI)	$\;\mathrm{SHDI}=-\sum_{m}^{i=1}\left(P_{i}{\mathrm{lnP}}_{i}\right)$	P_i_ = proportion of the landscape occupied by patch type (class) i.	SHDI > 0, without limit SHDI = 0 when the landscape contains only 1 patch (i.e., no diversity).	Shannon’s diversity index is a popular measure of diversity in community ecology, applied here to landscapes. Shannon’s index is more sensitive to rare patch types than Simpson’s diversity index.
	Shannon’s Evenness Index (SHEI)	$\mathrm{SHEI}=\frac{-\sum_{i=1}^{m}\left(P_{i}\ln P_{i}\right)}{\ln m}$	Pi= proportion of the landscape occupied by patch type (class) i. m = number of patch types (classes) present in the landscape, excluding the landscape border if present.	0 ≤ PLAND ≤ 100	Shannon’s evenness index is expressed such that an even distribution of area among patch types results in maximum evenness.

**Table A2:** Landscape pattern index analysis for each land use cover types.

		CA	PLAND	LPI	PD	ED	LSI	AI
Cropland	2000	4,521,294	33.5139	10.185	0.3903	19.9131	317.138	95.5389
	2005	4,479,107	33.1142	9.4578	0.373	20.5887	330.249	95.332
	2010	4,345,691	32.2123	7.4388	0.3737	20.4054	331.397	95.2444
	2015	4,305,303	31.8294	7.1532	0.3974	20.2421	331.096	95.2266
	2020	4,273,271	31.5771	3.5793	0.3859	21.4548	351.825	94.9076
Forest land	2000	7,522,174	55.7578	23.3248	0.0688	18.0137	223.423	97.5667
	2005	7,525,235	55.6343	23.2101	0.0695	18.4972	229.705	97.4985
	2010	7,510,385	55.6704	23.2349	0.0722	18.3296	227.517	97.5199
	2015	7,504,276	55.4797	22.6986	0.0809	18.1418	225.682	97.5391
	2020	7,476,049	55.2438	22.618	0.0809	19.2024	238.594	97.3927
Grassland	2000	563,287	4.1753	0.1258	0.0402	3.5096	159.475	93.6622
	2005	567,447	4.1952	0.1245	0.04	3.6098	163.97	93.507
	2010	552,914	4.0985	0.1091	0.0393	3.5068	160.654	93.5551
	2015	555,256	4.105	0.103	0.0416	3.4987	160.553	93.5734
	2020	548,622	4.054	0.1047	0.0406	3.6392	166.92	93.277
Built-up area	2000	285,397	2.1155	0.024	0.1193	2.9705	187.701	89.5083
	2005	296,981	2.1956	0.0253	0.1207	3.1102	193.191	89.4113
	2010	366,518	2.7168	0.0463	0.1276	3.5906	200.237	90.1199
	2015	437,021	3.2309	0.0737	0.1331	4.2058	215.339	90.267
	2020	468,242	3.46	0.0794	0.1356	4.6674	230.916	89.9154
Bare	2000	19,756.2	0.1464	0.0208	0.0013	0.0946	23.4168	95.2003
	2005	19,816.8	0.1465	0.0207	0.0014	0.0996	24.7945	94.9155
	2010	19,717.5	0.1462	0.0207	0.0011	0.0903	22.3863	95.4168
	2015	19,894.1	0.1471	0.0208	0.0012	0.0899	22.5558	95.402
	2020	19,284.5	0.1425	0.016	0.0012	0.0923	23.0756	95.2196

Note: CA—Class area; PLAND—Percent of landscape; LPI—Largest patch index; PD—Patch density; ED—Edge density; LSI—Landscape shape index; AI—Agglomeration index.
